# Insufficient Number of Controls for Low SARS-CoV-2 Seroprevalence: Are the Positive Rates Statistically Different between Pre-pandemic and Post-pandemic Samples?

**DOI:** 10.4269/ajtmh.22-0522

**Published:** 2022-10-31

**Authors:** Tomoko Fujitani, Kouji H. Harada

**Affiliations:** Department of Health Environmental Sciences Kyoto University Graduate School of Medicine Kyoto, Japan E-mail: kharada-hes@umin.ac.jp.

Dear Sir,

We read the article by Nguyen et al.^1^ in *The American Journal of Tropical Medicine and Hygiene* with great interest. The authors surveyed the seroprevalence of neutralizing antibodies against SARS-CoV-2 in Vietnam in 2020 and considered the positives in plaque reduction neutralization tests (PRNT) as SARS-CoV-2 infection. SARS-CoV-2 neutralizing antibodies were identified in two blood donors in samples collected in 2020 (*N* = 885; 0.23%; 95% confidence interval: 0.06–0.82).

We have comments on this finding. The PRNT is considered specific unless there is a cross-reactivity from neutralizing antibodies to other coronaviruses. Antibodies to seasonal common cold coronaviruses (CCC; 229E, NL63, HKU1, or OC43) might cross-react with SARS-CoV-2. A study reported that two of 40 CCC convalescent sera had inconclusive results in a SARS-CoV-2 micro-focus reduction neutralization test.^2^ Another study showed SARS-CoV-2 PRNT titers of 1:20 and 1:10 for OC43 and 229E convalescent sera, respectively.^3^

The authors conducted the PRNT for 46 pre-pandemic stored sera and found no positive result. The false-positive rate was 0%, but the 95% confidence interval was 0 to 7.7%. As already mentioned, PRNT for SARS-CoV-2 could show cross-reactivity to anti-CCC antibodies, and therefore, the false-positive rate should be determined accurately. In this case, the number of positive results should be statistically different between pre-pandemic (2017) and post-pandemic (2020) samples. To verify this point, we analyzed the rates by Fisher’s exact test (Table 1), which showed no significant difference in SARS-CoV-2 positivity between the two sample sets (*P* = 0.90). The number of controls is apparently too small to provide adequate power to evaluate the difference. In our previous study, 400 pre-pandemic samples were assayed by lateral flow assay and ELISA and showed 1.5% and 1.8% SARS-CoV-2 positives, respectively.^4^

**Table 1:** The number of positive and negative plaque reduction neutralization tests in blood donors (2017 and 2020)

	2017	2020
PRNT positive	0	2
PRNT negative	46	883

PRNT = plaque reduction neutralization tests.

*P* = 0.90 by Fisher’s exact test.

We think that the results in the paper by Nguyen et al. should be interpreted with caution. In particular, it should be noted that the seroprevalence in a low-prevalence situation of COVID-19 would be biased by the specificity of the test.
